# Poly(oligoethylene glycol methylether methacrylate-co-methyl methacrylate) Aggregates as Nanocarriers for Curcumin and Quercetin

**DOI:** 10.3390/polym17050635

**Published:** 2025-02-27

**Authors:** Michaila Akathi Pantelaiou, Dimitrios Vagenas, Stergios Pispas

**Affiliations:** 1 Theoretical and Physical Chemistry Institute, National Hellenic Research Foundation, 48 Vassileos Constantinou Ave., 11635 Athens, Greece; akathi39@gmail.com (M.A.P.); dimitrisv98@gmail.com (D.V.); 2 Department of Chemistry, National and Kapodistrian University of Athens, Panepistimiopolis Zografou, 15771 Athens, Greece

**Keywords:** amphiphilic copolymers, self-assembly, RAFT polymerization, nanocarriers, curcumin, quercetin

## Abstract

Amphiphilic statistical copolymers can be utilized for the formulation of nanocarriers for the drug delivery of insoluble substances. Oligoethylene glycol methylether methacrylate and methyl methacrylate are two biocompatible monomers that can be used for biological applications. In this work, the synthesis of linear poly(oligoethylene glycol methylether methacrylate-co-methyl methacrylate), P(OEGMA-co-MMA), and statistical copolymers via reversible addition fragmentation chain transfer (RAFT) polymerization is reported. P(OEGMA-co-MMA) copolymers with different comonomer compositions were synthesized and characterized by size exclusion chromatography (SEC), ^1^H-NMR, and ATR-FTIR spectroscopy. Self-assembly studies were carried out by the dissolution of polymers in water and via the co-solvent protocol. For the characterization of the formed nanoaggregates, DLS, zeta potential, and fluorescence spectroscopy (FS) experiments were performed. Such measurements delineate the association of copolymers into aggregates with structural characteristics dependent on copolymer composition. In order to investigate the drug encapsulation properties of the formed nanoparticles, curcumin and quercetin were loaded into them. The co-solvent protocol was followed for the encapsulation of varying concentrations of the two drugs. Nanocarrier formulation properties were confirmed by DLS while UV–Vis and FS experiments revealed the encapsulation loading and the optical properties of the drug-loaded nanosystems in each case. The maximum encapsulation efficiency was found to be 54% for curcumin and 49% for quercetin. For all nanocarriers, preliminary qualitive biocompatibility studies were conducted by the addition of FBS medium in the copolymer aqueous solutions which resulted in no significant interactions between copolymer aggregates and serum proteins. Novel nanocarriers of curcumin and quercetin were fabricated as a first step for the utilization of these statistical copolymer nanosystems in nanomedicine.

## 1. Introduction

Research in polymer science is of utmost importance as it deals with materials utilized in critical areas such as nanomedicine and biomedical engineering [1,2]. Step-growth and chain-growth polymerization techniques were the first to be developed. However, these methods produce polymers with high molar mass dispersity. More developments resulted in the introduction of the living/controlled polymerization mechanisms. Such techniques produce polymers with well-defined molecular characteristics and properties. These methods include, among others, the atom-transfer radical polymerization (ATRP), the nitroxide-mediated radical polymerization (NMP), and the reversible addition–fragmentation chain transfer (RAFT) polymerization technique [3]. Since 1998, when RAFT polymerization was first reported by CSIRO, research in the field has increased [4,5]. RAFT polymerization is a versatile technique that can produce polymeric products with complex architectures (block, star, and hyperbranched) [6]. A vast variety of monomers can be polymerized with precise control in molecular weight and distribution following rather simple polymerization procedures, easily adapted for upscaling. Furthermore, hybrid and functionalized polymers can be synthesized as well as bioconjugates [7].

Amphiphilic copolymers consist of hydrophilic and hydrophobic polymer domains. Due to the amphiphilic properties of the macromolecules, the polymeric chains can self-assemble in aqueous solution. Self-assembly occurs so that the hydrophobic part minimizes its contact with water resulting in the formation of core-shell morphologies with a hydrophobic core and a hydrophilic corona [8,9]. Poly(methyl methacrylate) is a well-studied hydrophobic polymer with many applications in various industries [10,11]. Its properties include mechanical and thermal stability, transparency, and biocompatibility while it has a low production cost [12,13]. Poly(oligoethylene glycol methylether methacrylate) is a hydrophilic, biocompatible, and thermoresponsive polymer. It is considered one of poly(ethylene glycol)’s alternatives [14]. OEGMA consists of methacrylate and ethylene oxide chains. Ethylene oxide chains influence the hydrophilicity of the polymer [15]. Recently, Wang et al. studied the synthesis of amphiphilic OEGMA-MMA copolymers and proved that they self-assemble in aqueous medium [16].

The self-assembly of amphiphilic polymers can result in the creation of polymeric nanocarriers. Nanocarrier morphologies include polyion complexes, micelles, nanogels, and polymersomes, among others. Various insoluble substances, such as dyes, natural molecules, and drugs, can be encapsulated covalently, electrostatically, or via the hydrophobic effect in the core or the corona of the nanoparticle [17,18]. The properties of these nanosystems can be precisely controlled, leading to biocompatible, efficient, stable, and stimuli-responsive nanocarriers [19]. Applications of these nanoparticles include drug and gene delivery for cardiovascular, neurodegenerative diseases, bacterial infections, and cancer. Also, nanocarriers have been utilized for bioimaging applications [18]. Curcumin is a natural constituent found in the herb *Curcuma longa* (L.) [20,21]. Its hydrophobic properties can be attributed to the two aromatic rings of the molecule [22]. Curcumin is used as an anti-inflammatory and antioxidant compound. Due to its hydrophobic property, it can enter into cancer cells and interact with signaling pathways, inhibiting cancer proliferation [22]. Apart from the pharmacological properties of curcumin, its structure and optical properties make it an ideal candidate for bioimaging applications [23]. A major drawback that curcumin faces is its low bioavailability and rapid clearance from the body [24]. The efficient encapsulation of curcumin into nanoformulations can solve this problem [21]. Furthermore, Wang et al., proved that upon encapsulation of curcumin in poly(lactic-co-glycolic) acid nanoparticles, the fluorescence is enhanced due to the aggregation-induced emission phenomenon (AIE) [25].

Another natural substance that has been actively investigated as a natural anticancer agent is quercetin [26]. Quercetin is a hydrophobic flavonoid found in fruits and vegetables presenting three aromatic rings with various hydroxyl groups [27]. Its structure makes it an antioxidant widely used as an anticancer and anti-inflammatory molecule [20,22]. Various studies have investigated the effectiveness of quercetin nanoformulations for the treatment of cancer [28]. Qureshi et al. encapsulated doxorubicin and quercetin in a methoxy poly(ethylene glycol) and poly(D, L-lactide-co-glycolide) nanoparticle [29]. Another innovative study by Nalinbenjapun et al. reported the synthesis of quercetin-conjugated chitosan for the fabrication of curcumin-loaded micelles [30]. Quercetin can also find applications in bioimaging as a fluorescent molecule. Upon encapsulation in polymeric nanosystems its emission is boosted as the aggregation-induced emission phenomenon occurs [31].

In this study, the encapsulation of curcumin and quercetin in P(OEGMA-co-MMA) nanocarriers is reported. Firstly, the synthesis of three polymers with different comonomer ratios was carried out by RAFT polymerization. Polymeric materials were characterized by NMR, ATR-FTIR, and SEC, while self-assembly studies were carried out by DLS, ELS, and FS spectroscopy in aqueous medium. In order to understand the effect of polymer composition on the loading efficiency of nanocarriers, the encapsulation of different loading ratios of curcumin and quercetin was performed by the co-precipitation method. The nanocarriers were characterized by UV-Vis, FS, and DLS. Finally, biocompatibility was tested by the addition of FBS medium in all samples.

## 2. Materials and Methods

### 2.1. Materials

For the synthesis of P(OEGMA-co-MMA), the monomers oligoethylene glycol methylether methacrylate (OEGMA) M_n_ = 500 g/mol and methyl methacrylate (MMA) were passed through purification columns with the inhibitor remover resins hydroquinone monomethyl ether (MEHQ) and butylated hydroxytoluene (BHT). All were obtained from Sigma-Aldrich (Athens, Greece). As a chain transfer agent (CTA), the 4-cyano-4-(phenylcarbonothioylthio) pentanoic acid (CPAD) was chosen from Sigma-Aldrich (Athens, Greece). The radical initiator 2,2-azobis(isobutyronitrile) (AIBN) was recrystallized from methanol. The solvents 1,4-dioxane, n-hexane, tetrahydrofuran (THF), and deuterated chloroform (CDCl_3_) were purchased from Sigma-Aldrich (Athens, Greece). Pyrene, curcumin, and quercetin utilized for the characterization and formulation of nanocarriers were received from Sigma-Aldrich (Athens, Greece).

### 2.2. Synthesis of Linear P(OEGMA-co-MMA)

**Figure 1:**
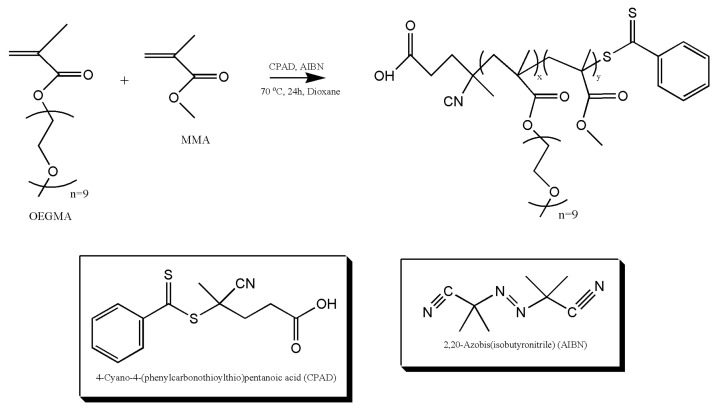
Copolymer synthesis scheme.

Three amphiphilic copolymers of the P(OEGMA-co-MMA) type with targeted %wt comonomer compositions of [70:30], [50:50], and [30:70], referred to as P1, P2, and P3, were synthesized by RAFT polymerization as depicted in Figure 1. For this purpose, the ratio of chain transfer agent to radical initiator was chosen to be 2:1, and the intended molecular weight of the final products is 20,000 g/mol. In a round-bottom flask, the CPAD, AIBN, and monomers were added and dissolved in 1,4-dioxane under stirring. The flask was sealed with a rubber septum and degassed via a nitrogen bubbler for 20 min. After, the flask was placed in an oil bath at 70 °C under stirring. The flask was left for 24 h in the oil bath as polymerization occurs at this stage. Then, the flask was cooled at −20 °C for 20 min. Afterwards the rubber septum was removed, and termination occurred. For the collection of resulting material, the precipitation of polymer in hexane took place. Any leftover material in the beaker was dissolved and collected with THF. The final polymeric product was dried in dynamic vacuum until the evaporation of the solvents.

### 2.3. Self-Assembly in Aqueous Solutions

The self-assembly-related experiments were conducted in an aqueous medium for the utilization of nanocarriers for biological applications. As the three different copolymer compositions were expected to provide different hydrophobicity to the copolymers, two distinct dissolution protocols were followed. In protocol A, aqueous solutions of polymer concentration 10^−3^ g/mL were created and left at the bench overnight in order for dissolution to occur. The second protocol was that of the co-solvent. For this reason, 10 mg of each copolymer was dissolved in 1 mL of THF for 1 h. Following that, each solution was injected into 10 mL of deionized water. The solutions were left under mild stirring for two hours at 60 °C. Then, they were kept under stirring overnight for the complete evaporation of THF. Any evaporated water was added the following day until reaching a 10 mL final volume.

### 2.4. Curcumin and Quercetin Nanocarrier Formulation

The co-solvent protocol was followed for the creation of loaded copolymer nanocarriers, as both curcumin and quercetin are hydrophobic. Polymer concentration was kept constant at 10^−3^ g/mL while different concentrations of curcumin and quercetin were investigated in order to achieve the maximum loading efficiency. For each formulation, the natural substance and copolymer were dissolved in THF for one hour. The rapid injection of solution in 10 mL of water followed. Temperature was set at 60 °C for two hours with mild stirring. Solutions were left at room temperature and stirred mildly overnight. Water was added until reaching a 10 mL final volume before measurements.

### 2.5. FBS Interaction with Nanoparticles

Protein interactions of FBS with nanoaggregates were tested in all samples. A solution of 1.5 mL of FBS/PBS (1:9 *v*/*v*) and 150 μL of each sample was prepared and left on the bench for one hour until DLS measurement.

### 2.6. Characterization Methods

#### 2.6.1. Size Exclusion Chromatography (SEC)

The molecular weight and distribution of each copolymer were determined by a Waters SEC chromatography system with THF as eluent (containing 5% triethylamine). The system is made of an isocratic Waters 1515 pump, µ-Styragel separation columns with pores of 10^2^–10^6^ Å, and a Waters 2414 refractive index detector set at 40 °C. The selected flow rate was 1 mL/min while chromatograms were analyzed by the Breeze v2.0 software.

#### 2.6.2. Proton Nuclear Magnetic Resonance Spectroscopy (^1^H-NMR)

Copolymer composition was determined by ^1^H-NMR spectroscopy. A copolymer solution of c = 10 mg/mL was created in each case. As a solvent, CDCl_3_ was utilized while tetramethylsilane was the internal reference. The spectrometer used was a Varian 300 (300 MHz) operated by Vjnmr software (OpenVnmrJ 1.1A). All spectra were analyzed with the MestReNova software v. 6.0.2 by MestReLabs (Santiago de Compostela, Spain).

#### 2.6.3. Attenuated Total Reflectance (ATR)-Fourier Transform Infrared (FTIR) Spectroscopy

A Bruker Equinox 55 spectrometer consisting of a single-bounce ATR diamond (Dura-Samp1IR II, SensIR Technologies, Danbury, CT, USA) was used for the measurements. The spectra were recorded utilizing a press as samples were in the solid state. For each measurement, 64 scans were performed with a resolution of 4 cm^−1^.

#### 2.6.4. Dynamic Light Scattering (DLS)

All samples were filtered prior to measurements with 0.45 µm hydrophilic PVDF filters. Nanoaggregate characterization was performed on an ALV/CGS-3 compact goniometer system (ALV GmbH, Hessen, Germany). The system is equipped with a JDS Uniphase He-Ne laser source (632.8 nm wavelength), a multi-τ correlator with 288 channels (ALV-5000/EPP), and an ALV/LSE-5003 control unit as the electronic interface system. For each sample, the average of five measurements was calculated at a 90° angle. The analysis of the results was done by the cumulants method and the CONTIN algorithm using software provided by the manufacturer. The CONTIN analysis of the time correlation functions revealed the distribution of relaxation times, τ. These values are utilized for the calculation of the diffusion coefficient D_app_ via the equation D_app_ = 1/τq^2^. The q value stands for the scattering vector. The Stokes-Einstein equation, R_h_ = k_B_T/6πη_0_D_app_, was used to determine the hydrodynamic radius, R_h_, at the maximum of the distribution peak. Here, k_B_ is the Boltzmann constant, T is the measurement temperature, and η_0_ is the viscosity of the medium [32]. All calculations were performed via the software provided.

#### 2.6.5. Electrophoretic Light Scattering (ELS)

Zeta potential measurements were conducted on the Nano Zeta Sizer (Malvern Panalytical, Malvern, UK). As a laser source, a 633 nm wavelength He–Ne laser was used. Each sample was measured 20 times at an angle of 173° and analyzed via the Smoluchowski equation. The average of all measurements is reported.

#### 2.6.6. Fluorescence Spectroscopy (FS)

Fluorescence spectra were measured on a NanoLog Fluorometer (Horiba Jobin Yvon, Kyoto, Japan). For excitation, a NanoLED 440 nm laser operated at 100 ps was used. A quartz cuvette with a 3 mL volume capacity was used. The dilution of samples occurred at a ratio of 1:10. For curcumin and quercetin excitation, the 425 nm wavelength was selected. The measurements for the determination of critical aggregate concentration (CAC) were carried out utilizing pyrene as the probe. CAC was calculated from the fluorescence intensity of the pyrene I_1_/I_3_ ratio. The excitation wavelength of pyrene measurements was set at 335 nm.

#### 2.6.7. UV–Vis Absorption Spectroscopy (UV–Vis)

Dilution conditions of a 1:10 ratio were also employed for absorption spectroscopy. Measurements were conducted on a Perkin–Elmer (Lambda 19, Waltham, MA, USA) UV–Vis–NIR spectrophotometer with quartz cuvettes.

## 3. Results and Discussion

### 3.1. P(OEGMA-co-MMA) Molecular Characterization

**Figure 2:**
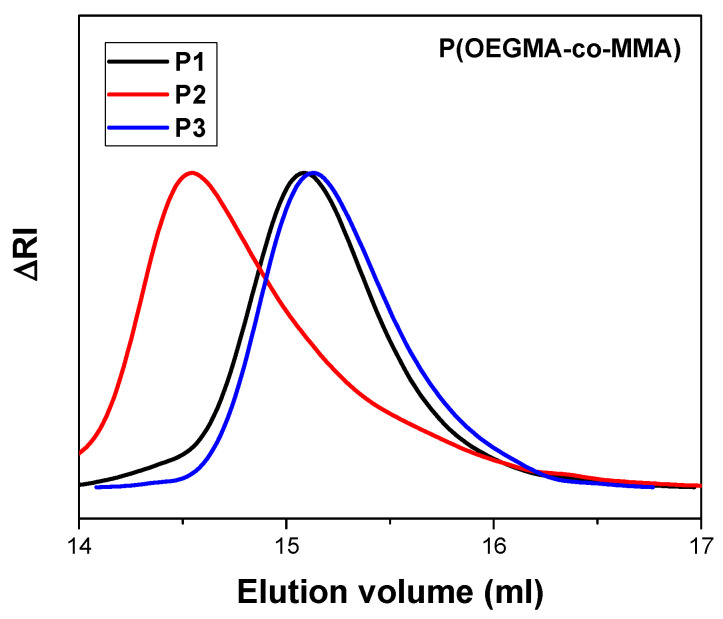
SEC chromatograms of the copolymers synthesized.

The three copolymers of different comonomer compositions were successfully synthesized and characterized by SEC chromatography. Molecular characteristics are presented in detail in Table 1. SEC chromatograms confirm the presence of unimodal peaks with polydispersity indexes under 1.3 as depicted in Figure 2. The results are in accordance with the literature as RAFT polymerization produces polymers with low polydispersity indexes [33]. All molecular weights are near 20,000 g/mol, which was the selected value for all syntheses. As reported by Akar et al., the distribution of comonomers in the polymeric chain of P(OEGMA-co-MMA) appears to be statistical [34].

**Figure 3:**
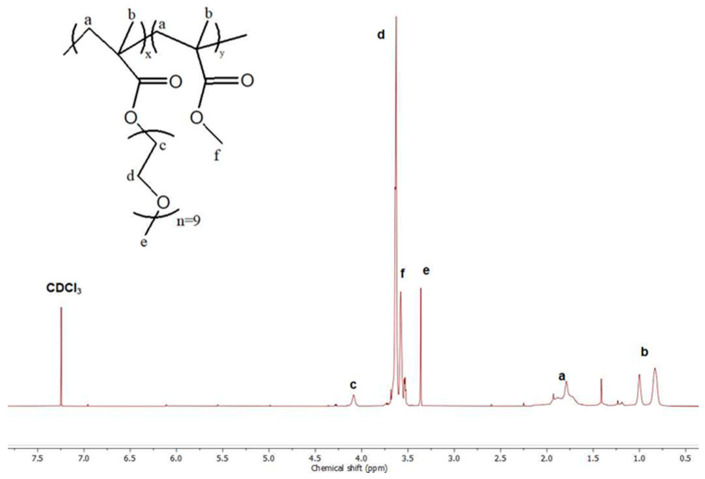
^1^H-NMR spectrum of P2 copolymer.

**Figure 4:**
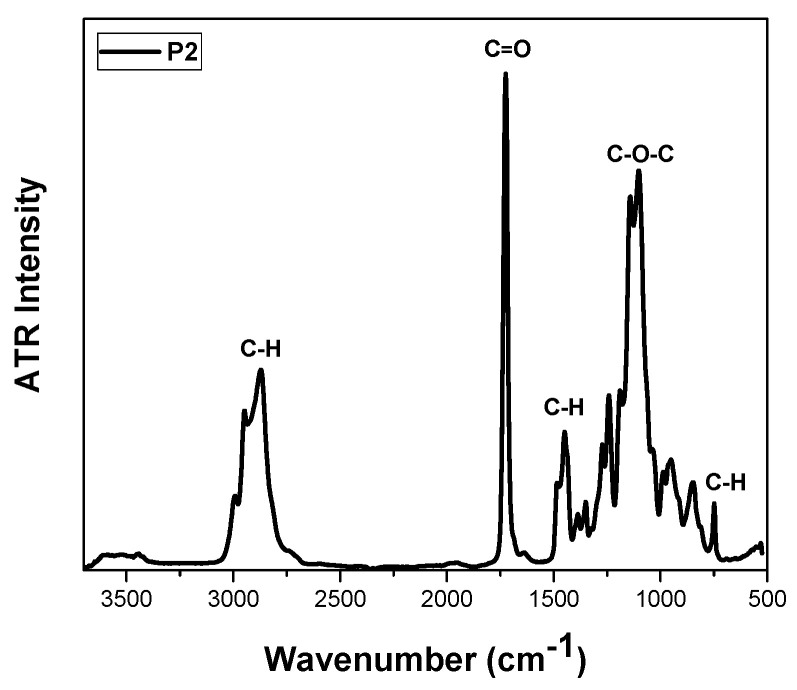
ATR-FTIR spectrum of P2 copolymer.

^1^H-NMR analysis revealed the chemical structure of polymeric products. Figure 3 presents the ^1^H-NMR spectrum of the P2 copolymer. The supplementary section contains ^1^H-NMR spectra of P1 and P3 (Figures S1 and S2). As presented in previous studies, peaks at 0.8 ppm and 1 ppm correspond to the –CH_3_ group of OEGMA and MMA segments. The peak at 3.3 ppm is attributed to the –CH_3_ group of OEGMA [34]. For the quantification of the weight composition of each comonomer, the integration of peaks at 0.8 and 3.3 ppm was implemented. Results are presented in Table 1. ATR-FTIR measurements also confirmed the presence of both monomers. The spectrum of P2 is presented in Figure 4. The characteristic peak of 1727 cm^−1^ can be attributed to the C=O bond, which appears in both comonomers. Furthermore, the stretching at 1104 cm^−1^ can be assigned to the C-O-C group of both segments [35].

### 3.2. Self-Assembly and Critical Aggregation Concentration in Aqueous Solutions

**Figure 5:**
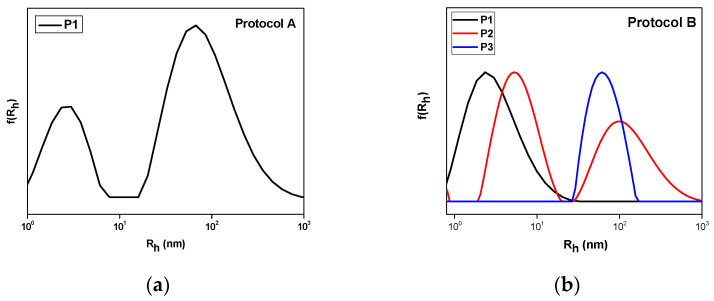
DLS measurements for (**a**) copolymer P1 via direct dissolution protocol A and (**b**) co-solvent protocol B for all copolymers studied.

The self-assembly of copolymers was carried out in deionized water as the experiments also concerned biological applications of the nanosystems. The OEGMA_500_ monomer is hydrophilic due to its ethylene glycol units, and thus P1 was expected to be more hydrophilic due to its higher percentage of OEGMA segments. The dissolution of copolymers following protocol A in water proved that only P1 is hydrophilic. P2 and P3 could not be dissolved directly in water. In order to confirm the amphiphilic nature of all copolymers, dissolution via the co-solvent protocol B was carried out. All polymers form nanoparticles and the DLS results are presented in Figure 5 and Table 2. ELS measurements confirmed the low surface charge of the nanoaggregates (Table 2).

**Figure 6:**
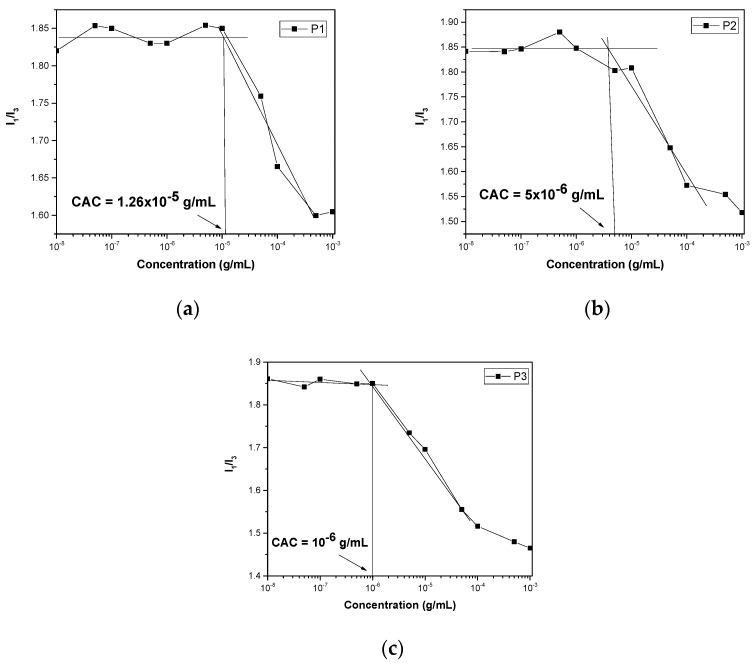
CAC determination for (**a**) P1; (**b**) P2; and (**c**) P3 amphiphilic copolymers.

For sample P1, different nanoparticles were created by the two protocols. Protocol A resulted in the formation of single-chain nanoparticles of 2.5 nm radius and larger nanostructures of 80 nm, while the co-solvent protocol produced only single-chain nanoaggregates. Intensity appears to be lower in the case of protocol B as nanoparticles are smaller. This behavior should be attributed to the insolubility of MMA segments of the copolymers in water. Comparing the self-assembly of P1, P2, and P3 by the co-solvent protocol, it appears that as the MMA content increases, the peaks shift to larger dimensions while the mass is also increased. Surface charge is near to zero for all samples as both comonomers are non-ionic. For the determination of the critical aggregation concentration, pyrene was utilized as the fluorescent probe. Pyrene tends to be encapsulated in the hydrophobic domains of the aggregates [36]. A range of eleven concentrations of copolymer was measured by FS (Figure 6). A plateau is observed in smaller concentrations while a change in slope occurs at the beginning of aggregate formation. Analysis in the intersection of the two fitted straight lines, where the slope changes, revealed that CAC tends to decrease from 1.26 × 10^−5^ g/mL to 1 × 10^−6^ g/mL as the hydrophobic MMA content increased in the copolymers.

#### 3.2.1. Curcumin Encapsulation

**Figure 7:**
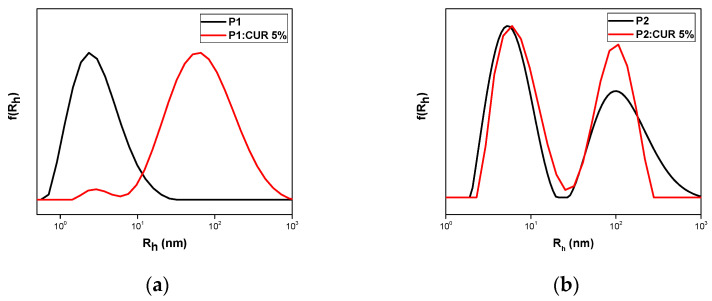

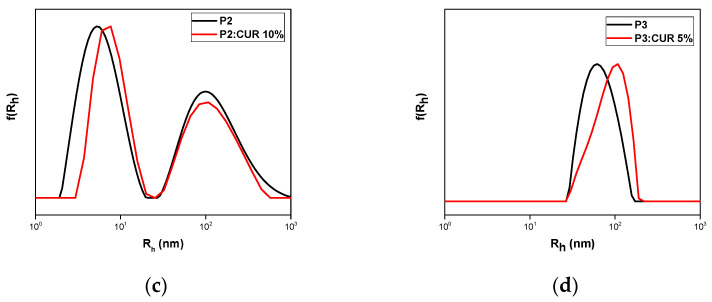
DLS measurements of curcumin nanocarriers one day after preparation: (**a**) P1:CUR 5%; (**b**) P2:CUR 5%; (**c**) P2:CUR 10%; and (**d**) P3:CUR 5%.

The hydrophobic substance curcumin was loaded in nominal concentrations of 5 and 10 wt% in each copolymer. Out of all nanoformulations prepared, only the P1:CUR 5%, P2:CUR 5%, P2:CUR10%, and P3:CUR 5% remained stable for 20 days with no presence of precipitation in the solutions initially prepared. DLS measurements confirmed the formation of nanocarriers as depicted in Figure 7. Table 3 presents the encapsulation characteristics of the stable nanocarriers one day after preparation. Polymeric nanocarrier P1:CUR 5% presents an increase in radius to 61 nm compared to pure P1. Small variations in radius were observed in all other samples. Maximum encapsulation loading was achieved for sample P2:CUR 10%.

**Figure 8:**
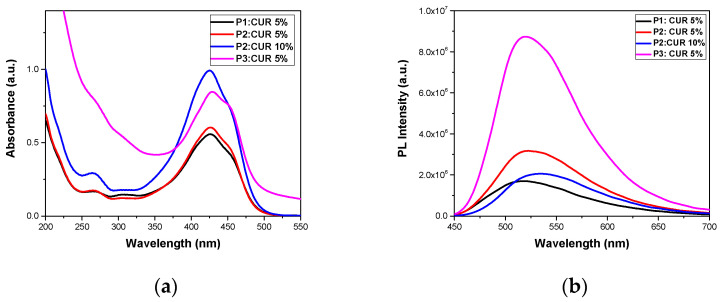
Characterization of nanoaggregates one day after preparation by (**a**) UV–Vis and (**b**) FS spectroscopy.

Apart from DLS measurements, UV–Vis and FS characterization confirmed the successful encapsulation of curcumin in all copolymer aggregates. Figure 8 depicts the UV–Vis and FS spectra of all nanosystems. Pure curcumin in THF was measured in different concentrations by UV–Vis technique as a reference for the quantification of encapsulation efficiency and loading. In Supplementary Figure S3, curcumin fluorescence is also presented with maximum fluorescence emission at 504 nm [37]. All nanocarriers exhibit absorption in the range of 425–428 nm as reported in the literature [38]. Fluorescence emission was measured in the range of 518–535 nm. The increase in the emission intensity that the nanocarriers present can be attributed to the aggregation-induced emission phenomenon that was previously reported in the literature by Wang et al. [25]. Curcumin, as a hydrophobic molecule, tends to aggregate in the hydrophobic domains of the polymeric nanoparticles [39]. It appears that the encapsulation of curcumin inside the hydrophobic regions stabilizes the molecule, which is the main cause of the AIE phenomenon. As sample P3:CUR 5% consists of more MMA segments, stabilization occurs in the hydrophobic core, and that is the reason for the enhanced emission and the redshift that is observed in this case.

**Figure 9:**
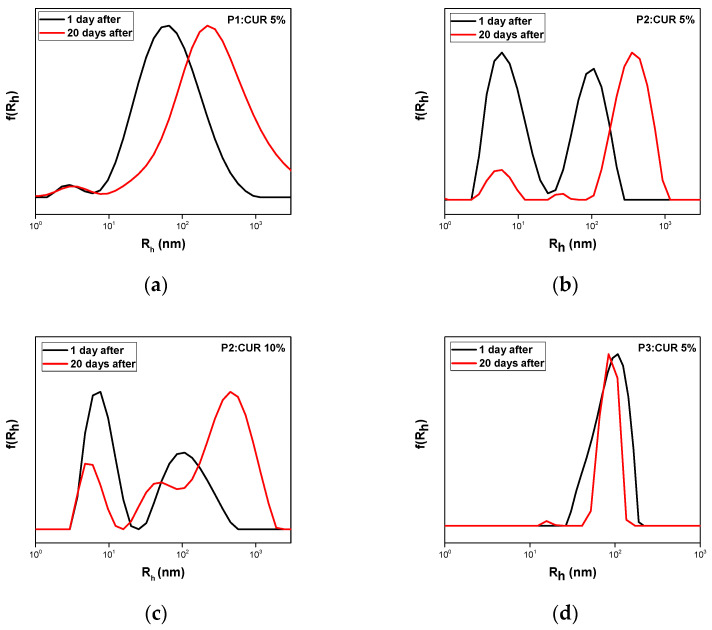
DLS stability measurements of CUR loaded nanocarriers one day and 20 days after preparation: (**a**) P1:CUR 5%; (**b**) P2:CUR 5%; (**c**) P2:CUR 10%; and (**d**) P3:CUR 5%.

In order to prove the thermodynamic stability of the obtained loaded copolymer nanoparticles, measurements were repeated 20 days after their preparation. No precipitation was observed in the vials. Measurements are presented in Figure 9 and Table 3. All nanosystems appear to be stable with no formation of larger aggregates, although there is a shift in all peaks to higher dimensions apart from sample P3:CUR 5%. This can be attributed to the incorporation of more polymeric chains in the aggregates in order to attain a stable structure in solution. The system seems to be reorganized with time. Nanocarriers also retained their photophysical properties for 20 days as reported by UV–Vis and FS measurements shown in Figure S4. UV–Vis confirms the presence of the characteristic absorption peaks of curcumin for all of the nanocarriers. Fluorescence emission intensity was also stable, indicating the applicability of such nanoformulations in bioimaging.

#### 3.2.2. Quercetin Encapsulation

**Figure 10:**
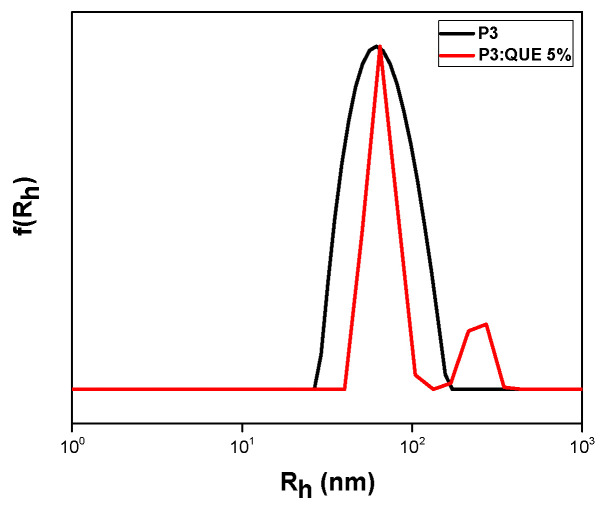
DLS measurement of P3:QUE 5% nanocarrier formulation one day after preparation.

Quercetin was also loaded in the P(OEGMA-co-MMA) nanoaggregates. Different loading concentrations were tested for all copolymers, but only one sample retained its stability over 20 days. This may be related to the different chemical structure of quercetin in comparison to curcumin and the different interactions developing between the drugs and the copolymers at the molecular level. Figure 10 depicts the DLS measurement of the P3:QUE 5% sample one day after preparation. The characteristics of the nanoformulations are presented in Table 4.

**Figure 11:**
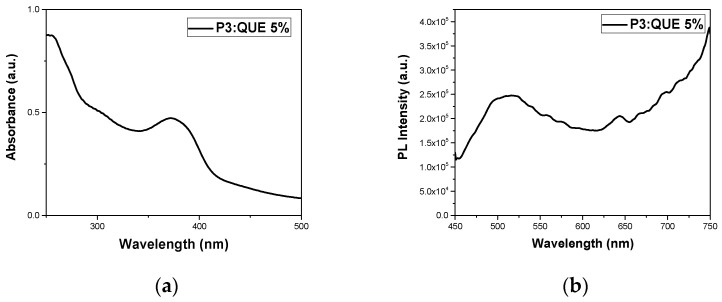
Quercetin nanoformulation one day after preparation: (**a**) UV–Vis and (**b**) FS spectra.

**Figure 12:**
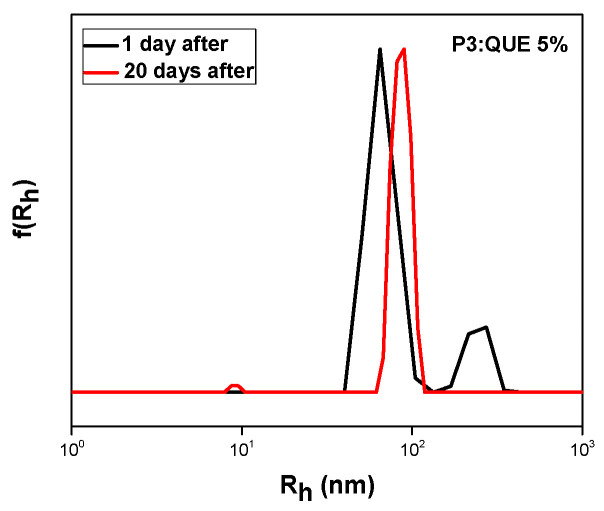
DLS stability measurement of nanocarrier P3:QUE 5% 20 days after preparation.

Two different peaks appeared in sample P3:QUE 5%. One at 66 nm, which is the dominant one, and another at 241 nm. It can be observed that since quercetin is hydrophobic, only the P3 copolymer can encapsulate this drug molecule, as it has the highest MMA content compared to the other copolymers. UV–Vis and fluorescence experiments of pure quercetin in THF were also carried out in different concentrations for the determination of the calibration curve. The fluorescence emission of quercetin at 485 nm is depicted in Figure S5. The specific nanocarrier has also been characterized by UV–Vis and FS spectroscopy. The corresponding spectra are presented in Figure 11. The nanocarrier appears to present an absorption peak at 372 nm while the emission is observed at 516 nm [40]. Nanocarrier emission intensity was boosted compared to pure quercetin due to the AIE phenomenon [31]. All experiments were also carried out 20 days after the preparation of the formulation, revealing the stability of the nanosystem as it is depicted by Figure 12 and Figure S6 and Table 4. There was no precipitation in the solution. DLS confirmed the presence of nanocarriers with a change in radius from 66 nm to 81 nm. The previously observed peak at 241 nm on day 1 was not visible. It can be assumed that these changes may be attributed to structural rearrangements occurring in the mixed copolymer/QUE nanosystem over time.

### 3.3. FBS Interactions with P(OEGMA-co-MMA) Nanoaaggregates

**Figure 13:**
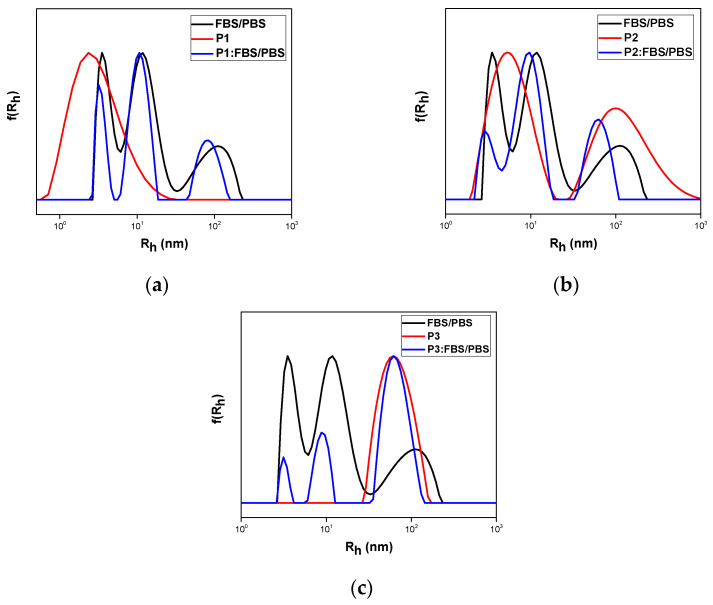
FBS interaction with copolymer aggregates (**a**) P1, (**b**) P2, and (**c**) P3 studied by DLS measurements.

**Figure 14:**
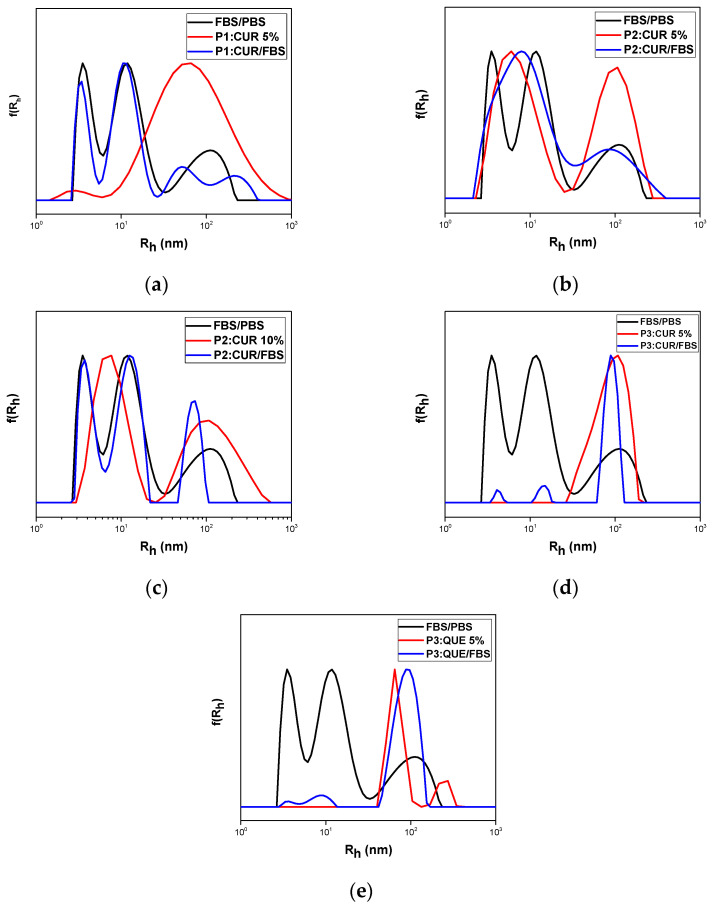
FBS interaction with nanocarriers (**a**) P1:CUR 5%; (**b**) P2:CUR 5%; (**c**) P2:CUR10%; (**d**) P3:CUR 5%; and (**e**) P3:QUE 5%, respectively, as revealed by DLS.

All copolymer aggregates as well as the loaded nanocarriers were tested for their interactions with FBS medium. This is a first step for testing qualitatively the biocompatibility and stability of the nanosystems in a blood-stimulating environment. One day after preparation, the addition of FBS took place according to the experimental protocol described above, followed by DLS measurements. FBS peaks appeared in all measurements according to literature [17]. Both comonomers are biocompatible and OEGMA segments forming the corona of the aggregates are expected to show protein repelling properties similar to PEG chains. The results confirm the biocompatibility and stability of the nanosystems in the presence of serum proteins, as there was no formation of significantly larger aggregates in any of the samples tested compared to neat FBS medium. As far as the interactions of FBS with neat copolymer aggregates are concerned, the nanoaggregate peaks were not altered, as can be seen in Figure 13, indicating no aggregation of the nanoparticles in the protein-containing medium. The obtained size distributions after the addition of FBS are presented in Figure 14.

## 4. Conclusions

We reported the synthesis of P(OEGMA-co-MMA) amphiphilic statistical copolymers by RAFT polymerization and their self-assembly in nanoscale aggregates. The two well-known and important comonomers for nanomedicine, OEGMA and MMA, were utilized for this purpose. The molecular characterization of the copolymers and self-assembly studies by DLS and FS proved the formation of biocompatible nanoaggregates. The encapsulation of curcumin and quercetin was achieved in the formed aggregates, and with quercetin it was harder to produce stable loaded nanosystems, showcasing the effects of drug chemical structure on the encapsulation process. The maximum encapsulation loading of curcumin proved to be higher, ca. 4.8%, than that of quercetin, ca. 2.4%. Stability was confirmed for all of the nanocarriers over a period of 20 days. Photophysical studies on the loaded copolymer aggregates confirmed the AIE phenomenon for the two hydrophobic drugs, highlighting the possibility of using them in bioimaging assays. Preliminary biocompatibility tests in FBS solutions also confirmed the suitability of these nanocarriers for biological applications. Further experiments need to be carried out in order to explore the biocompatibility of the formed nanoparticles. The obtained results could be a road map for applications of nanosized amphiphilic statistical copolymer aggregates in image-guided drug delivery and bioimaging for cancer treatment.

## Figures and Tables

**Table 1:** Molecular characteristics of the synthesized P(OEGMA-co-MMA) amphiphilic copolymers.

Sample	M_w_^a^ (×10^4^ g/mol)	M_w_/M_n_^a^	OEGMA ^b^ (wt%)	MMA ^b^ (wt%)
P1	1.73	1.15	62	38
P2	2.56	1.29	56	44
P3	1.58	1.12	19	81

^a^: Determined by SEC. ^b^: Determined by H^1^-NMR.

**Table 2:** DLS and ELS results of P(OEGMA-co-MMA) nanoaggregates.

Sample	Int_90_ (Kcps)	PDI	R_h, cont_ (nm)	Zeta Potential (mV)
P1-Protocol A	60	0.35	2.5 80	-
P1-Protocol B	24	0.45	2.9	3.6
P2	79	0.49	5.7 121	2.3
P3	384	0.17	65	−7.3

**Table 3:** Curcumin encapsulation results.

Sample	Curcumin Maximum Loading wt%	R_h, cont_ (nm) Day 1	R_h, cont_ (nm) Day 20	Encapsulation Efficiency %	Encapsulation Loading %
P1	5	61	242	42	2.1
P2	5	6 107	5 37 351	54	2.6
P2	10	8 113	6 41 450	50	4.8
P3	5	85	85	39	1.9

**Table 4:** Sample P3:QUE 5% characterization results.

Sample	Quercetin Maximum Loading wt%	R_h, cont_ (nm) Day 1	R_h, cont_ (nm) Day 20	Encapsulation Efficiency %	Encapsulation Loading %
P3	5	66 241	81	49	2.4

## Data Availability

Data produced in this study are included in the manuscript and the Supplementary Materials.

## Supplementary Materials

The following supporting information can be downloaded at https://www.mdpi.com/article/10.3390/polym17050635/s1, Figure S1: ^1^H-NMR spectrum of P1 copolymer; Figure S2: ^1^H-NMR spectrum of P3 copolymer; Figure S3: Curcumin fluorescence measurement in THF (c_CUR_ = 1 mg/mL); Figure S4: Stability measurements of nanocarriers 20 days after preparation: (a) UV–Vis and (b) FS spectra; Figure S5: FS spectrum of quercetin in THF(c_QUE_ = 1 mg/mL); Figure S6: Stability measurements of nanocarrier P3:QUER 5% 20 days after preparation: (a) UV–Vis and (b) FS spectra.
