# Supplementary material for: Poly(oligoethylene glycol methylether methacrylate-co-methyl methacrylate) Aggregates as Nanocarriers for Curcumin and Quercetin
Source: Polymers (Basel). 2025 Feb 27;17(5):635. doi: 10.3390/polym17050635 (PMC11902823; doi:10.3390/polym17050635)
Supplement: Supplementary file 1 [file polymers-17-00635-s001.zip › polymers-3477634-supplementary.pdf]

Supplementary Materials: Poly(oligoethylene glycol methylether methacrylate-co-methyl methacrylate) aggregates as nanocarriers for curcumin and quercetin

Michaila Akathi Pantelaiou[1,2], Dimitrios Vagenas[1,2] and Stergios Pispas[1,*]

[1] Theoretical and Physical Chemistry Institute, National Hellenic Research Foundation, 48 Vassileos Constantinou Ave., 11635 Athens, Greece; akathi39@gmail.com (M.A.P.), dimitrisv98@gmail.com (D.V.), pispas@eie.gr (S.P.)
[2] Department of Chemistry, National and Kapodistrian University of Athens, Panepistimiopolis Zografou, 15771 Athens, Greece
* Correspondence: pispas@eie.gr

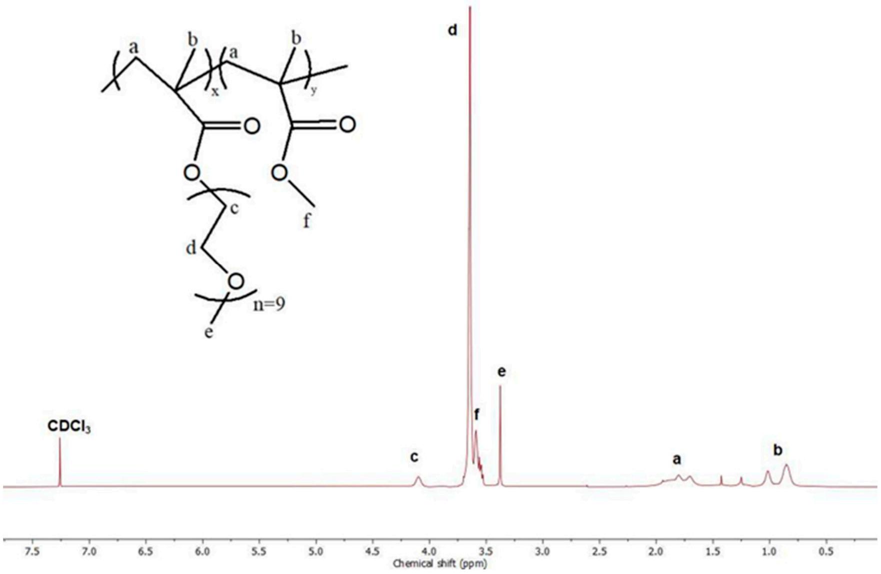

Figure S1. ^{1}H-NMR spectrum of P1 copolymer.

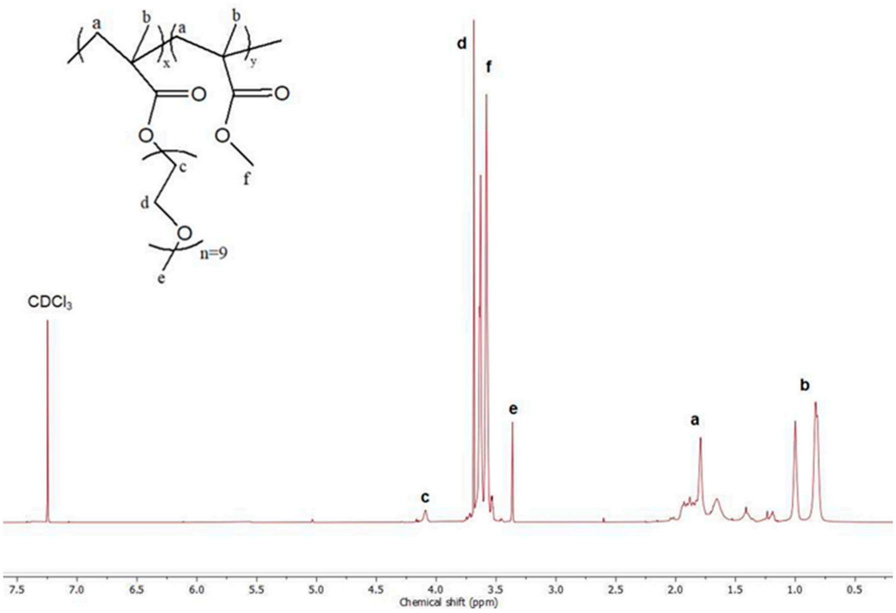

Figure S2. ^{1}H-NMR spectrum of P3 copolymer.

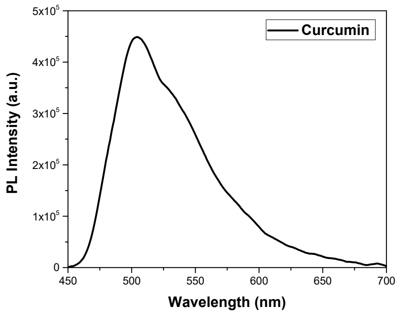

Figure S3. Curcumin fluorescence measurement in THF (c_{CUR}= 1mg/mL).

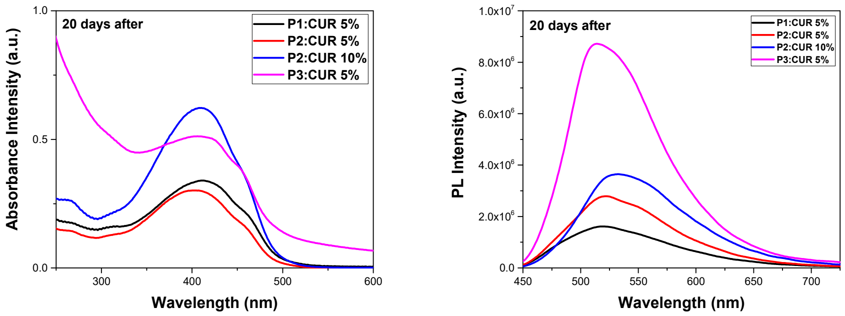

(a) **(b)**

Figure S4. Stability measurements of nanocarriers20 days after preperation; (a) UV-Vis; (b) FS spectra.

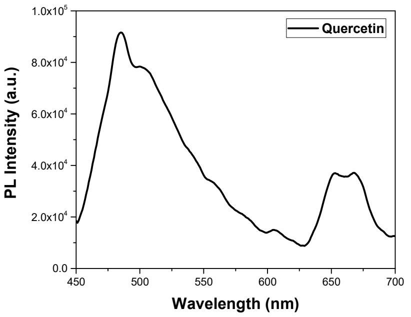

Figure S5. FS spectrum of quercetin in THF (c_{QUE} = 1 mg/mL).

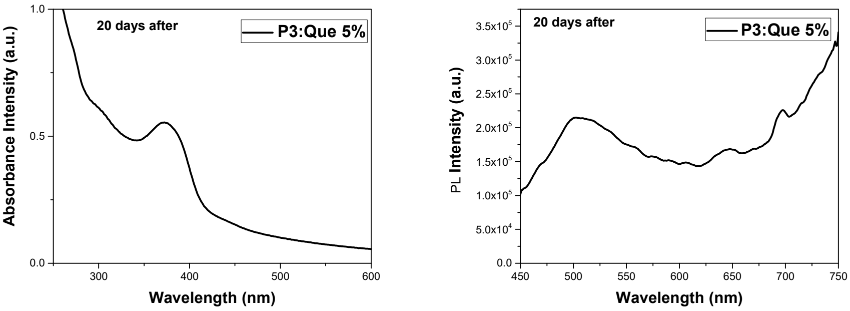

(a) **(b)**

Figure S6. Stability measurements of nanocarrier P3:QUER 5% 20 days after preperation; (a) UV-Vis; (b) FS spectra.
